# A fully-annotated imagery dataset of sublittoral benthic species in Svalbard, Arctic

**DOI:** 10.1016/j.dib.2021.106823

**Published:** 2021-01-30

**Authors:** Andrius Šiaulys, Evaldas Vaičiukynas, Saulė Medelytė, Sergej Olenin, Aleksej Šaškov, Kazimieras Buškus, Antanas Verikas

**Affiliations:** a Marine Research Institute, Klaipeda University, Klaipeda, Lithuania; b Faculty of Informatics, Kaunas University of Technology, Kaunas, Lithuania; c Faculty of Electrical and Electronics Engineering, Kaunas University of Technology, Kaunas, Lithuania; d Faculty of Mathematics and Natural Sciences, Kaunas University of Technology, Kaunas, Lithuania

**Keywords:** Underwater imagery, Mosaicking, ROV, Drop-down camera, Machine vision, Image segmentation, Semantic segmentation

## Abstract

Underwater imagery is widely used for a variety of applications in marine biology and environmental sciences, such as classification and mapping of seabed habitats, marine environment monitoring and impact assessment, biogeographic reconstructions in the context of climate change, etc. This approach is relatively simple and cost-effective, allowing the rapid collection of large amounts of data. However, due to the laborious and time-consuming manual analysis procedure, only a small part of the information stored in the archives of underwater images is retrieved. Emerging novel deep learning methods open up the opportunity for more effective, accurate and rapid analysis of seabed images than ever before.

We present annotated images of the bottom macrofauna obtained from underwater video recorded in Spitsbergen island's European Arctic waters, Svalbard Archipelago. Our videos were filmed in both the photic and aphotic zones of polar waters, often influenced by melting glaciers. We used artificial lighting and shot close to the seabed (<1 m) to preserve natural colours and avoid the distorting effect of muddy water. The underwater video footage was captured using a remotely operated vehicle (ROV) and a drop-down camera. The footage was converted to 2D mosaic images of the seabed. 2D mosaics were manually annotated by several experts using the Labelbox tool and co-annotations were refined using the SurveyJS platform.

A set of carefully annotated underwater images associated with the original videos can be used by marine biologists as a biological atlas, as well as practitioners in the fields of machine vision, pattern recognition, and deep learning as training materials for the development of various tools for automatic analysis of underwater imagery.

## Specifications Table

**Table:** 

Subject	Marine biology, Computer Vision and Pattern Recognition
Specific subject area	Underwater imagery, mosaicking, semantic segmentation, machine vision.
Type of data	Video, image, annotations, table
How data were acquired	Underwater video footage was filmed with a remotely operated vehicle (ROV) and a drop-down camera. Video samples were converted into 2D mosaic images of the seabed. 2D mosaics were manually annotated using the Labelbox tool and co-annotations refined using the SurveyJS platform.
Data format	Raw video (.avi), 2D mosaics (.jpg), annotated images (.png), tables (.csv)
Parameters for data collection	Original data were collected by filming the seabed at 3–65 meter depths in 3–10 min transects with 50 fps (frames per second). Video samples were prepared in 10–30 s segments for stitching of a 2D mosaic and had 3–5 fps.
Description of data collection	The dataset consists of three directories: video samples, video mosaics and annotated categories with/without background. 47 video samples and 47 resulting 2D mosaics with corresponding annotations (masks and mask overlays) for 2242 objects in 12 categories.
Data source location	The following bays of Svalbard archipelago: Adriabukta (77.000100, 16.192216) Burgerbukta (77.057108, 16.007882) Borebukta (78.38859, 14.28120) Dahlbrebukta (78.566666, 12.368533) Eidembukta (78.360133, 12.779950) Gipsvika (78.42591, 16.52873) St. Johnsfjord (78.506766, 12.931066) Trygghamna (78.254050, 13.761500) Country: Norway
Data accessibility	Data identification number: DOI: 10.17632/mmzb4hhptc.1 Direct URL to data: http://dx.doi.org/10.17632/mmzb4hhptc.1

## Value of the Data

• The dataset presents annotated images of Arctic bottom macrofauna derived from the underwater video. The dataset can be useful both as a biological atlas and training material for automatic segmentation solutions. Seabed imagery data can be used for multiple purposes in marine biology and environmental sciences, for example: benthic habitat classification and mapping, marine environmental monitoring, impact assessment, biogeographical reconstructions in the context of climate change, etc.
• A set of carefully annotated underwater images, linked to source videos, is of great value for both marine biologists and researchers as well as practitioners working in the fields of Machine Vision, Pattern Recognition, Machine Learning, and Deep Learning.
• The data will be used for the development of methods and tools for automatic identification of biological categories in underwater imagery, semantic image segmentation, object detection, and automatic characterization of the seabed. The data might be used for validation of various machine vision applications (i.e. automatic identification of biological organisms in underwater imagery), educational purposes (i.e. training material for marine scientists) and other tasks.
• There is a lack of annotated underwater imagery datasets with just a few recently published cases [1], [2], [3] featuring coarse categories from various camera angles. Liu and Fang [1] collected 2537 images with 16 categories (nautilus, squid, plant, coral, fish, jellyfish, dolphin, sea lion, Syngnathus, turtle, starfish, shrimp, octopus, seahorse, person, stone). SUIM dataset [2] contains 1635 images with 7 categories (human diver, aquatic plant or sea-grass, wreck or ruins, robot, reef and invertebrates, fish and vertebrates, sea-floor or rock). Martin-Abadal et al. [3] annotated 483 images of *Posidonia oceanica* meadows.
• The most similar dataset to ours in terms of biological accuracy and seabed aspect is the coral reef study by King et al. [4], where 9511 cropped images of one object representing 10 categories (*Acropora palmata, Orbicella* spp., Siderastrea, *Porites astreoides, Gorgonia ventalina*, sea plume, sea rod, algae, rubble, sand) were prepared. More coral reef transects exist [5], [6] even a web-based solution for coral reef analysis – CoralNet [7].
• Our video was captured in both the photic and aphotic zones of polar waters, often in the vicinity of melting glaciers. We used artificial lighting and shot close to the seabed to preserve natural colours and avoid the distorting effect of turbid waters.

## 1 Data Description

**Fig. 1:**
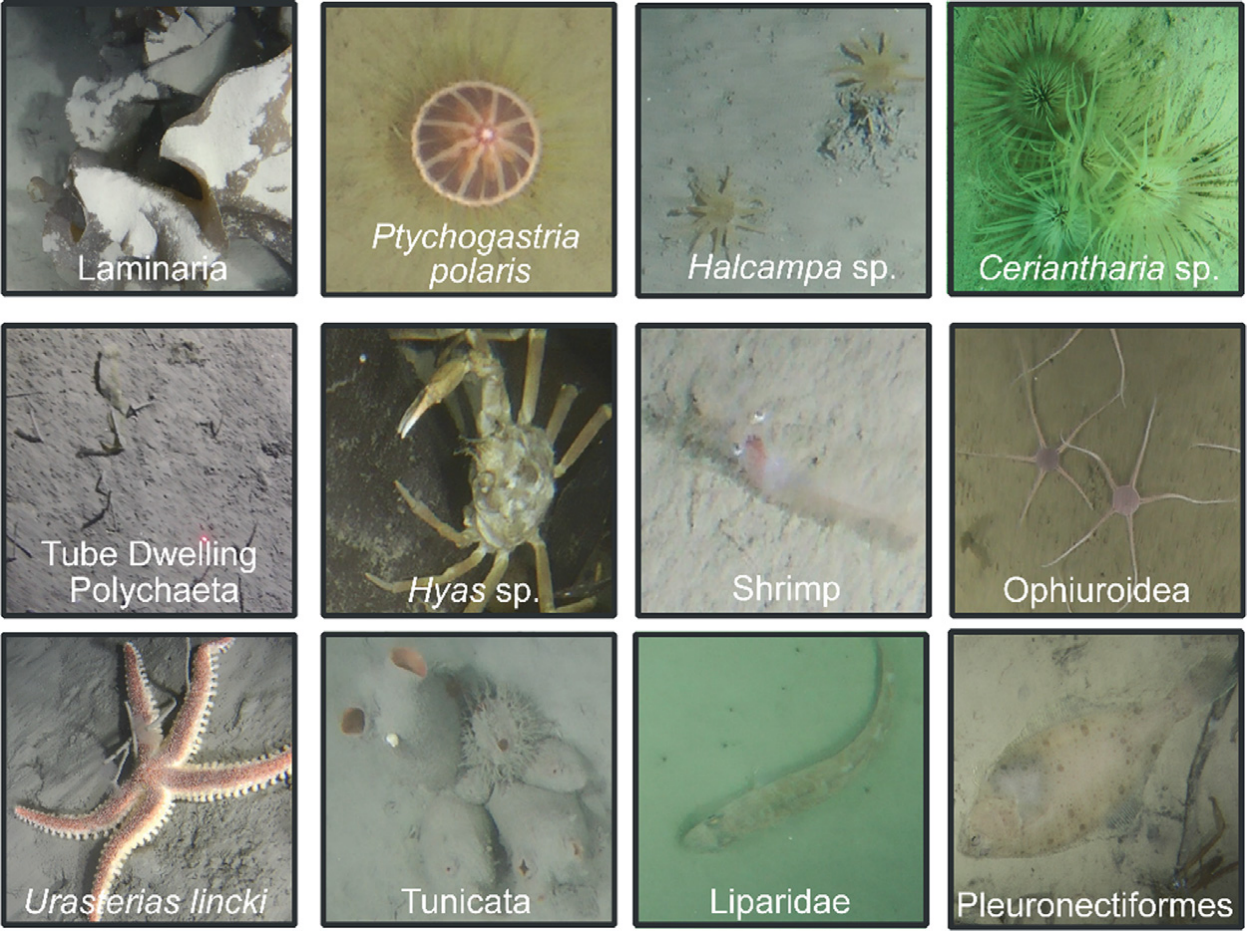
Typical examples of 12 different categories (biological taxons) targeted in the annotation process.
c) Annotations. The annotation process, where four experts performed manual pixel-wise segmentation (see Fig. 2) and mask refinement survey (see Fig. 3), resulted in 2242 annotated objects with the most frequent category – Ophiuroidea. The annotation outcome is summarized by listing mosaics for each category label (see Table 1) and listing category labels for each mosaic (see Table 2). The example of 2D mosaic, mosaic with masked objects and their overlay is shown in Fig. 4.

We present visual data of bottom macrofauna filmed in the sublittoral of European Arctic – Svalbard. Some of the areas (Burgerbukta, Borebukta, Dahlbrebukta, St. Johnsfjorden, Trygghamna) are in the vicinity of melting glaciers; others are in ice-free areas (Adriabukta, Eidembukta, Gipsvika). The dataset [8] consists of three types of data:

a) Video samples. In total, 22 min 51 s of video footage was filmed and split into 10–30 s segments, resulting in 47 video samples; frame rate was reduced to 3–5 fps.
b) 2D mosaics. All video samples were converted into still images (video mosaics), that were manually analysed by marine biologists – specialists in the Arctic biota, who identified visible biological objects at the lowest possible taxonomic level. Twelve taxons were targeted for annotation (see Fig. 1): brown alga – kelp *Laminaria* sp*.*, benthic trachymedusa *Ptychogastria polaris,* burrowing anemone *Halcampa* sp., tube anemone *Ceriantharia* sp., tube-dwelling Polychaeta, spider crab *Hyas* sp., Shrimps, brittle stars Ophiuroidea, sea star *Urasterias lincki*, sea squirts Tunicata, snailfishes Liparidae and flatfishes Pleuronectiformes.

**Fig. 2:**
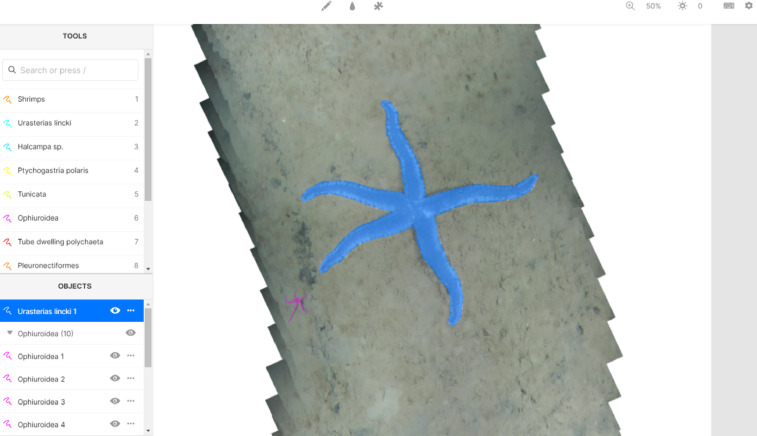
The graphical user interface of *Labelbox* annotation tool for mosaic B6_0409_30s: all possible configured labels for the selected annotation are visible in the TOOLS section (each category with different color of the mask); annotated category instances are registered in the OBJECTS section; the canvas area shows an annotated brittle star (purple mask) and selected sea star *Urasterias lincki* (blue mask) categories. (For interpretation of the references to color in this figure legend, the reader is referred to the web version of this article.)

**Fig. 3:**
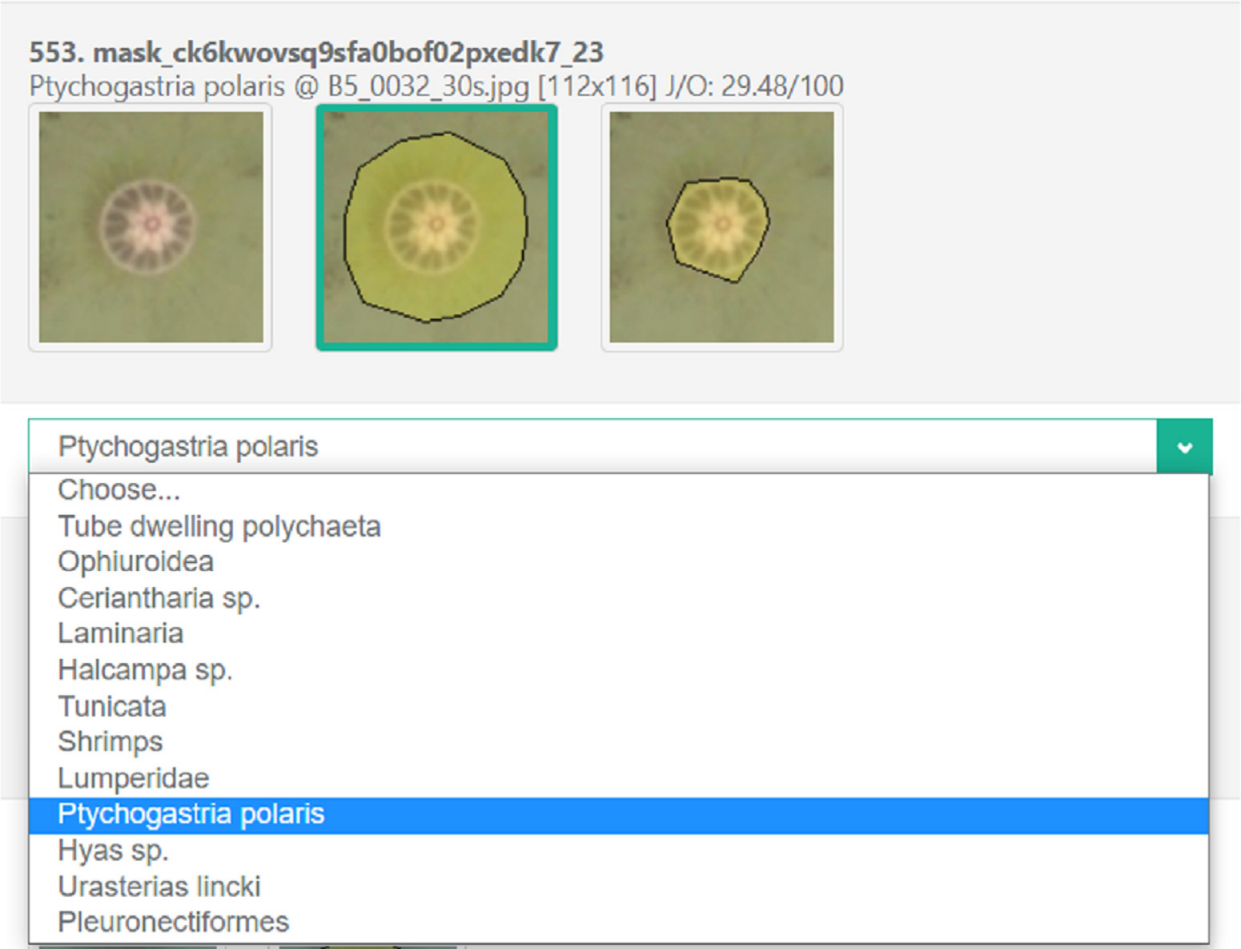
The view of a generated survey for the expert choice of co-annotated objects using the *SurveyJS* platform. Question structure: image picker with potential masks and dropdown for the category choice.

**Table 1:** Mosaics by label. 2242 objects distributed by the category label, starting with the most frequent one. Column “Mosaics” indicates the total count of mosaics containing at least one object of a category in question.

Label	Objects	Mosaics	Details
Ophiuroidea	974	11	(578) B5_0102_30 s, (252) B5_0032_30 s, (46) B6_0215_30 s, (40) B6_0311_30 s, (25) B6_0409_30 s, (18) B6_0040_30 s, (6) B7_0143_30 s, (3) B2_0458_27 s, (3) B3a_0536_14 s, (2) B1_0332_30 s, (1) B7_0438_30s
Tube dwelling polychaeta	890	28	(138) SJ(2)_1140_30 s, (101) HB03_0524_30 s, (81) G3_0928_30 s, (73) G3_0601_30 s, (68) G3_0821_30 s, (65) G3_0857_30 s, (59) G3_0705_30 s, (45) G3_0100_30 s, (37) G3_0458_30 s, (34) B7_0143_30 s, (32) SJ(2)_1109_10 s, (24) SJ(2)_0536_30 s, (21) HB03_0424_30 s, (20) B4_1509_30 s, (15) T1_0956_30 s, (13) B7_0438_30 s, (11) B6_0215_30 s, (10) B6_0040_30 s, (8) G3_0234_30 s, (8) HB03_0328_30 s, (7) B5_0102_30 s, (4) B6_0311_30 s, (4) G4_0035_30 s, (4) T1_0556_30 s, (4) T1_0732_30 s, (2) B5_0032_30 s, (1) B1_0332_30 s, (1) B3a_0536_14s
*Ceriantharia* sp.	233	12	(64) SJ(1)_0216_30 s, (41) B4_1509_30 s, (33) SJ(1)_0324_30 s, (26) SJ(1)_0250_30 s, (21) SJ(1)_0738_30 s, (16) SJ(2)_0536_30 s, (9) SJ(2)_1140_30 s, (7) B7_0438_30 s, (6) B7_0237_30 s, (5) D1(2)_1144_30 s, (4) B7_0143_30 s, (1) E2_0335_30s
*Laminaria* sp.	63	12	(19) D1(1)_0928_30 s, (13) G3_0100_30 s, (7) G3_0234_30 s, (7) SJ(1)_0738_30 s, (4) D1(1)_0855_30 s, (3) D1(1)_0822_30 s, (3) SJ(2)_0536_30 s, (2) B7_0237_30 s, (2) B7_0438_30 s, (1) B4_1509_30 s, (1) B7_0143_30 s, (1) SJ(2)_1140_30s
*Halcampa* sp.	49	3	(24) E2_0453_30 s, (13) E2_0235_30 s, (12) E2_0335_30s
Shrimps	29	5	(10) HB03_0424_30 s, (6) HB03_0328_30 s, (5) HB02_0322_30 s, (4) HB03_0524_30 s, (4) HB04_0318_30s
Liparidae	19	8	(7) B1_0432_30 s, (3) B1_0332_30 s, (2) B2_0458_27 s, (2) G3_0458_30 s, (2) HB04_0318_30 s, (1) D1(2)_1144_30 s, (1) E4_0215_30 s, (1) HB03_0524_30s
Tunicata	15	7	(5) T1_0556_30 s, (4) B7_0438_30 s, (2) T1_0732_30 s, (1) B6_0215_30 s, (1) D1(1)_0928_30 s, (1) SJ(1)_0250_30 s, (1) T1_0956_30s
*Hyas* sp.	11	8	(2) D1(1)_0855_30 s, (2) G3_0100_30 s, (2) G3_0601_30 s, (1) D1(1)_0822_30 s, (1) E2_0453_30 s, (1) E4_0215_30 s, (1) G3_0234_30 s, (1) G4_0035_30s
*Urasterias lincki*	11	7	(3) B3a_0536_14 s, (3) B6_0311_30 s, (1) B6_0040_30 s, (1) B6_0215_30 s, (1) B6_0409_30 s, (1) HB03_0328_30 s, (1) SJ(2)_1109_10s
*Ptychogastria polaris*	6	3	(4) B5_0102_30 s, (1) B2_0458_27 s, (1) B5_0032_30s
Pleuronectiformes	5	5	(1) AD02_0735_30 s, (1) G3_0100_30 s, (1) G3_0234_30 s, (1) G4_0035_30 s, (1) HB03_0424_30s

**Table 2:** Labels by mosaic. 2242 objects distributed by mosaic name, starting from the most crowded. Column “Categories” indicates how many unique categories were annotated for the mosaic in question.

Mosaic	Objects	Categories	Details
B5_0102_30s	589	3	(578) Ophiuroidea, (7) Tube dwelling polychaeta, (4) Ptychogastria polaris
B5_0032_30s	255	3	(252) Ophiuroidea, (2) Tube dwelling polychaeta, (1) Ptychogastria polaris
SJ(2)_1140_30s	148	3	(138) Tube dwelling polychaeta, (9) Ceriantharia, (1) Laminaria
HB03_0524_30s	106	3	(101) Tube dwelling polychaeta, (4) Shrimps, (1) Liparidae
G3_0928_30s	81	1	(81) Tube dwelling polychaeta
G3_0601_30s	75	2	(73) Tube dwelling polychaeta, (2) Hyas
G3_0821_30s	68	1	(68) Tube dwelling polychaeta
G3_0857_30s	65	1	(65) Tube dwelling polychaeta
SJ(1)_0216_30s	64	1	(64) Ceriantharia
B4_1509_30s	62	3	(41) Ceriantharia, (20) Tube dwelling polychaeta, (1) Laminaria
G3_0100_30s	61	4	(45) Tube dwelling polychaeta, (13) Laminaria, (2) Hyas, (1) Pleuronectiformes
B6_0215_30s	59	4	(46) Ophiuroidea, (11) Tube dwelling polychaeta, (1) Tunicata, (1) Urasterias lincki
G3_0705_30s	59	1	(59) Tube dwelling polychaeta
B6_0311_30s	47	3	(40) Ophiuroidea, (4) Tube dwelling polychaeta, (3) Urasterias lincki
B7_0143_30s	45	4	(34) Tube dwelling polychaeta, (6) Ophiuroidea, (4) Ceriantharia, (1) Laminaria
SJ(2)_0536_30s	43	3	(24) Tube dwelling polychaeta, (16) Ceriantharia, (3) Laminaria
G3_0458_30s	39	2	(37) Tube dwelling polychaeta, (2) Liparidae
SJ(1)_0324_30s	33	1	(33) Ceriantharia
SJ(2)_1109_10s	33	2	(32) Tube dwelling polychaeta, (1) Urasterias lincki
HB03_0424_30s	32	3	(21) Tube dwelling polychaeta, (10) Shrimps, (1) Pleuronectiformes
B6_0040_30s	29	3	(18) Ophiuroidea, (10) Tube dwelling polychaeta, (1) Urasterias lincki
SJ(1)_0738_30s	28	2	(21) Ceriantharia, (7) Laminaria
SJ(1)_0250_30s	27	2	(26) Ceriantharia, (1) Tunicata
B7_0438_30s	27	5	(13) Tube dwelling polychaeta, (7) Ceriantharia, (4) Tunicata, (2) Laminaria, (1) Ophiuroidea
B6_0409_30s	26	2	(25) Ophiuroidea, (1) Urasterias lincki
E2_0453_30s	25	2	(24) Halcampa, (1) Hyas
D1(1)_0928_30s	20	2	(19) Laminaria, (1) Tunicata
G3_0234_30s	17	4	(8) Tube dwelling polychaeta, (7) Laminaria, (1) Hyas, (1) Pleuronectiformes
T1_0956_30s	16	2	(15) Tube dwelling polychaeta, (1) Tunicata
HB03_0328_30s	15	3	(8) Tube dwelling polychaeta, (6) Shrimps, (1) Urasterias lincki
E2_0335_30s	13	2	(12) Halcampa, (1) Ceriantharia
E2_0235_30s	13	1	(13) Halcampa
T1_0556_30s	9	2	(5) Tunicata, (4) Tube dwelling polychaeta
B7_0237_30s	8	2	(6) Ceriantharia, (2) Laminaria
B1_0432_30s	7	1	(7) Liparidae
B3a_0536_14s	7	3	(3) Ophiuroidea, (3) Urasterias lincki, (1) Tube dwelling polychaeta
D1(2)_1144_30s	6	2	(5) Ceriantharia, (1) Liparidae
D1(1)_0855_30s	6	2	(4) Laminaria, (2) Hyas
T1_0732_30s	6	2	(4) Tube dwelling polychaeta, (2) Tunicata
HB04_0318_30s	6	2	(4) Shrimps, (2) Liparidae
B1_0332_30s	6	3	(3) Liparidae, (2) Ophiuroidea, (1) Tube dwelling polychaeta
B2_0458_27s	6	3	(3) Ophiuroidea, (2) Liparidae, (1) Ptychogastria polaris
G4_0035_30s	6	3	(4) Tube dwelling polychaeta, (1) Hyas, (1) Pleuronectiformes
HB02_0322_30s	5	1	(5) Shrimps
D1(1)_0822_30s	4	2	(3) Laminaria, (1) Hyas
E4_0215_30s	2	2	(1) Hyas, (1) Liparidae
AD02_0735_30s	1	1	(1) Pleuronectiformes

**Fig. 4:**
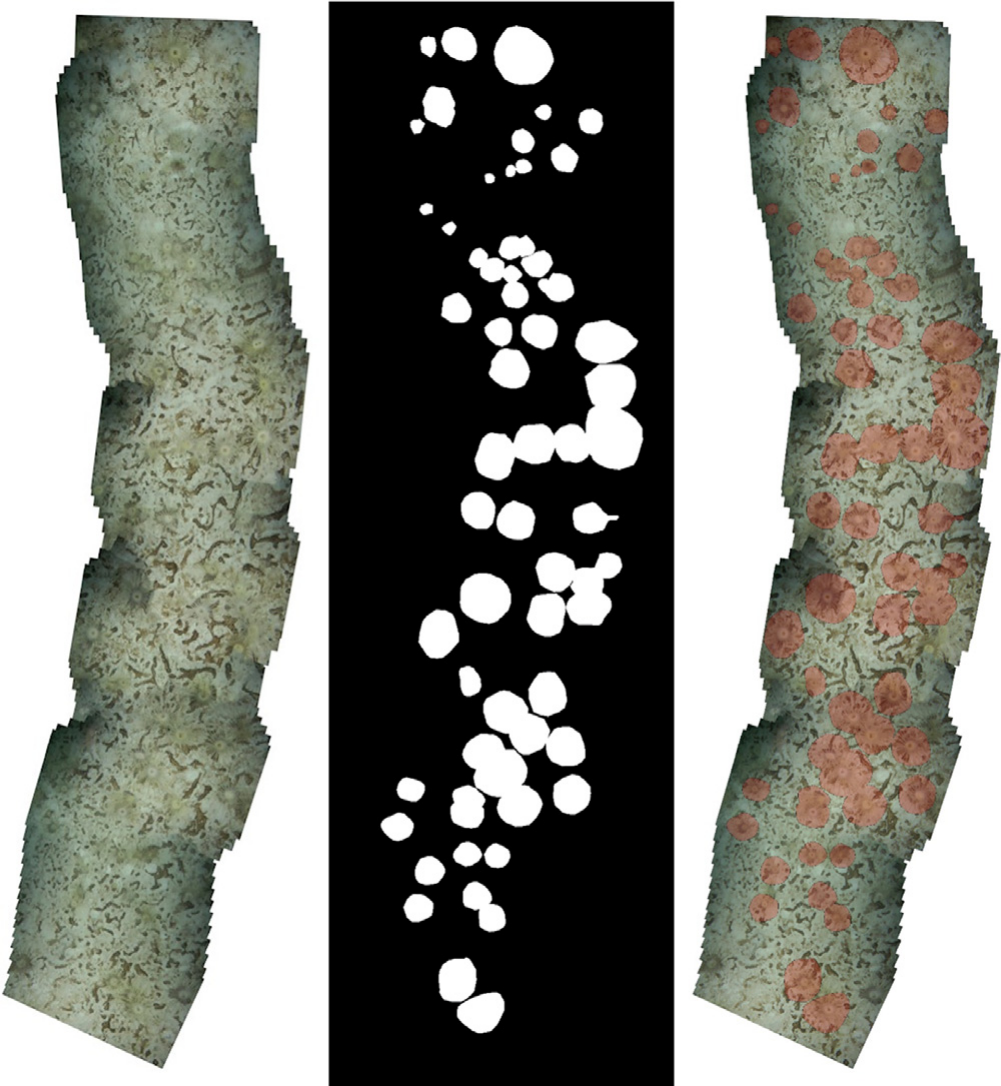
An example of 2D mosaic (left) with masked objects (center) and their overlay (right).

The labels of files indicate site, transect, a part of raw video from which video sample was cropped and the duration of a video sample. I.e., file named *B1_0332_30* *s.jpg*, where *B* stands for Borebukta bay (AD – Adriabukta, B – Borebukta, D – Dahlbrebukta, E – Eidembukta, G – Gipsvika, HB – Burgerbukta, SJ – St. Johnsfjorden, T – Trygghamna), *1* – transect number, *0332* – 3 min 32 s start time from a raw video, *30* *s* – length of video sample from which this mosaic file was made.

## 2 Experimental Design, Materials and Methods

### 2.1 Underwater video

Underwater video data were acquired with a ROV, equipped with a low-resolution analogue camera on a tilted unit for navigation, and a primary camera. The primary camera was mounted vertically, has 3 CCD, Full HD (1920 × 1080) resolution, high-quality Leica Dicomar lenses and 10x optical zoom. The primary camera lighting system consists of 16 bright LED in 4 × 4 stations. The ROV was used in Borebukta, Dahlbrebukta, Eidembukta, Gipsvika, St. Johnsfjord and in Trygghamna. A Drop-down camera was equipped with an analogue camera of 700 TV lines (TVL) resolution for live view and a digital camera (Panasonic HX-A500) that recorded the material at high resolution (1280  ×  720 px) on a memory card. The drop-down camera was used in Adriabukta and Burgerbukta. During the filming, camera speed was about 1 knot to avoid motion blur, and camera altitude over the seabed was 0.4–0.5 m, to ensure optimal lighting conditions. Stations near glaciers had very turbid water because of the inflow of glacial meltwater. At those stations, colours were slightly washed out due to light scattering on the suspended particles, but the imagery was still useful.

### 2.2 2D seabed mosaics

Video mosaicking is a process of converting a video sample into a single still image containing overlapping video frames. For the pre-mosaicking process raw videos were divided into 10–30 s video segments. Frame size was reduced, and the frame rate was lowered to 3–5 fps to shorten computing time. Each frame was enhanced for more accurate pair-wise registration and video mosaics were produced using original non-enhanced video footage and pair-wise registration data. Algorithms for video mosaicking have been developed by Rzhanov et al. [9], [10]. Taxonomic identification of benthic species was carried out with specialists’ help using a digital catalog, in which more than 40 biological (fish, benthic invertebrates, algae, etc.) and physical (stones, substrate, burrows, footprints, etc.) categories were identified. For simplicity, we decided to select 12 most prominent ones for annotation. No image post-processing was considered for a stitched mosaic and we would like to note that a large diversity of potentially useful water effect removal methods exist: from enhancement-based to restoration-based and even deep-learning-based post-processing [11].

### 2.3 Mosaic annotation

Prepared 47 large 2D mosaics were uploaded to the online collaborative annotation platform *Labelbox* [12]. A new project was created by configuring the default editor (video, image, and text annotation) to have 12 categories (termed as OBJECTS in the interface) and inviting the team members to join. All mosaics were inspected and identified objects were segmented by four different marine biology experts using the polygon tool (see Fig. 2). Since all the experts had all mosaics available, there was an intentional overlap between many segmented objects. The annotation results with URL links to mosaics and generated masks were downloaded in .json and .csv formats.

### 2.4 Mask refinement

Expert annotations, downloaded from *Labelbox*, were later processed using the R language script to form a survey for all masks (both overlapping between experts and unique) in .json format. Correctly formated .json survey was uploaded to the *SurveyJS* platform [13] for serving and collecting expert responses on each annotated object (see Fig. 3 for a survey question example). Resulting .json structure for an example survey with a single question is detailed in Table 3.

**Table 3:** Example JSON structure for a single survey question. More questions would be created by repeating “picker-dropdown” sequence inside elements array. Survey logic for dropdown element was configured so that it becomes visible only after mask is selected. JSON code could be copied and pasted into JSON Editor tab at https://surveyjs.io/create-survey and then previewed live in Test Survey tab.

Survey part details	JSON code
Header with page name	{ "pages": [ { "name": "Ptychogastria polaris (19)", "elements": [
Image picker to choose the best mask	{ "type": "imagepicker", "name": "mask_ck6kwovsq9sfa0bof02pxedk7_23″, "description": "Ptychogastria polaris @ B5_0032_30 s.jpg [112×116] J/O: 29.48/100″, "choices": [ { "value": "ck6kwovsq9sfa0bof02pxedk7_23″, "imageLink": "https://i.imgur.com/t3UHOy1.png" }, { "value": "ck8in1b2510ry0z7omx85fbdy", "imageLink": "https://i.imgur.com/wwI0EVI.png" }, { "value": "ckdbjusl10myh0yaj5jeygj6z", "imageLink": "https://i.imgur.com/XtKjqct.png" } ], "imageHeight": 116, "imageWidth": 112 },
Dropdown field to confirm or change the assigned category	{ "type": "dropdown", "name": "label_ck6kwovsq9sfa0bof02pxedk7_23″, "visibleIf": "{mask_ck6kwovsq9sfa0bof02pxedk7_23} notempty and {mask_ck6kwovsq9sfa0bof02pxedk7_23} 〈〉 'ck6kwovsq9sfa0bof02pxedk7_23′", "titleLocation": "hidden", "hideNumber": true, "defaultValue": "Ptychogastria polaris", "choices": [ "Tube dwelling polychaeta", "Ophiuroidea", "Ceriantharia sp.", "Laminaria", "Halcampa sp.", "Tunicata", "Shrimps", "Lumperidae", "Ptychogastria polaris", "Hyas sp.", "Urasterias lincki", "Pleuronectiformes" ] }
Footer with additional info	], "title": "Ptychogastria polaris", "description": "19 masks @ B2_0458_27 s.jpg, B5_0102_30 s.jpg, B5_0032_30 s.jpg, D1(1)_0928_30 s.jpg, E2_0335_30 s.jpg" } ] }

Post-processing of annotation results was as follows:

1. Find objects segmented only by a single expert.
2. Find objects segmented by several experts simultaneously. Create a new synthetic mask using a union of two masks with the highest overlap.
3. For each object cut-out its view from mosaic to get a background image.
4. For each mask of the object create an overlay to get overlayed images.
5. Upload background and overlayed images to a free image hosting service *imguR*.
6. For each object make a survey question using image picker and dropdown (see Table 3).
7. Upload the generated .json structure to the *SurveyJS* platform for survey serving (see Fig. 3);
8. Share a survey link with experts and ask them to fill out the survey, where they could:
  a. discard an object if all masks look inappropriate;
  b. choose the best mask for an object using an image picker;
  c. check an assigned category and change it using dropdown if needed.
9. Download survey results and choose the best mask using majority voting.

A few questions where each expert has chosen a different candidate mask were reviewed together to arrive at the consensus. There were also some questions where one mask was chosen by 2 experts and another mask was also chosen by 2 experts. This kind of tie was resolved by preferring a synthetic mask (if it existed among the choices made) or choosing between the two selected masks at random.

## CRediT Author Statement

**Andrius Šiaulys**: Writing- Original draft preparation, Data collection, Annotation, Data curation, Survey; **Evaldas Vaičiukynas**: Writing- Reviewing and Editing, Methodology, Mask refinement; **Saulė Medelytė**: Selection of taxons, Mosaicking, Annotation, Survey; **Sergej Olenin**: Data collection, Annotation, Conceptualization, Survey; **Aleksej Šaškov**: Data collection, Annotation, Survey; **Kazimieras Buškus**: Editing, Data curation; **Antanas Verikas**: Conceptualization, Supervision.

## Declaration of Competing Interest

None.
